# Determination of Ethanol Concentration in Kombucha Beverages: Single-Laboratory Validation of an Enzymatic Method, First Action Method 2019.08

**DOI:** 10.1093/jaoacint/qsaa122

**Published:** 2020-09-26

**Authors:** Ruth Ivory, Elaine Delaney, David Mangan, Barry V McCleary

**Affiliations:** Megazyme, Bray Business Park, Southern Cross Rd, Bray A98 YV29, Ireland

## Abstract

Kombucha is a fermented, lightly effervescent sweetened black or green tea drink. It is marketed as a functional beverage based on its proposed health benefits. Kombucha is produced by fermenting tea using a “symbiotic colony of bacteria and yeast” (SCOBY). Kombucha is marketed as a non-alcoholic beverage, however due to the production process employed, there is a high possibility that the Kombucha products will contain low levels of ethanol. Kombucha is sold in a raw and unpasteurized form and, if kept at temperatures above 4 °C, the possibility exists that it will continue to ferment, producing ethanol. This possibility of continued fermentation may lead to an increase in ethanol content from levels below 0.5%ABV at time of production to higher levels at time of consumption. Thus, there is a potential for levels rising to greater than 0.5%ABV, the threshold for certification as a non-alcoholic beverage. It is essential that Kombucha manufacturers have the capacity to accurately and quickly test for ethanol in their products.  The Ethanol Assay Kit is an enzymatic test kit developed by Megazyme for the determination of ethanol in a variety of samples. The kit has been validated in a single laboratory for use with Kombucha fermented drinks, fruit juices, and low-alcohol beer samples. The commercially available Ethanol Assay Kit (Megazyme catalogue no. K-ETOH) contains all components required for the analysis. Quantification is based on the oxidation of ethanol to acetaldehyde by alcohol dehydrogenase and further oxidation of acetaldehyde by acetaldehyde dehydrogenase with conversion of NAD^+^ to NADH. The single laboratory validation (SLV) outlined in this document was performed on a sample set of eight different commercial Kombucha products purchased in Ireland, a set of five Cerilliant aqueous ethanol solutions, two BCR low-alcohol beer reference materials, two alcohol-free beer samples, and two fruit juice samples against SMPR 2016.001 (1). Parameters examined during the validation included Working range, Selectivity, Limit of Detection (LOD), Limit of Quantification (LOQ), Trueness (bias), Precision (reproducibility and repeatability), Robustness, and Stability. The Ethanol Assay is a robust, quick and easy method for the measurement of ethanol in Kombucha. Our data suggests this method is also reliable for similar matrices, such as low-alcohol beer and fruit juice. The assay meets all requirements set out in in AOAC SMPR 2016.001.

##  

### Scope of Method

- *Target Analyte*—Ethanol; CAS Registry No. 64-17-5
- *Matrices*—For this validation, K-ETOH was tested with the following matrices:
  - Eight commercial kombucha samples (Samples K1–K8).
    K1; Ginger-flavored Raw Kombucha. Ingredients: Filtered Water, Green Tea, Sugar, Kombucha Culture, Grated Ginger
    K2; Blueberry-flavored Raw Kombucha. Ingredients: Filtered Water, Green Tea, Sugar, Kombucha Culture, Blueberry Juice
    K3; Green Tea-flavored Raw Kombucha. Ingredients: Water, Golden Cane Sugar, Sencha Green Tea, Citrus Tisane (Apple Bits, Grapes, Citrus Peels, Rosehips, Hibiscus, Liquorice, Natural Flavor), Ginger Juice, Pai Mu Tan White Tea, Kombucha Culture
    K4; Lemongrass and Ginger-flavored Raw Kombucha. Ingredients: Water, Golden Cane Sugar, Sencha Green Tea, Ginger Juice (3%), Lemongrass (2%), Kombucha Culture
    K5; Citrus-flavored Raw Kombucha. Ingredients: Sparkling Water, Kombucha Culture (Filtered Water, Black Tea Extract, Green Tea Extract, Natural Flavor), Filtered Water, Cane Sugar, Bacillus coagulans LactoSpore MTCC 5856, Ginger Extract, Orange Extract, Lemon Extract, Black Tea Extract, Black Tea Essence, Caffeine (Green Coffee Bean Extract), Green Tea Extract, Rosemary Extract, Lime Extract, Stevia Leaf Extract
    K6; Ginger-flavored Raw Kombucha. Ingredients: Sparkling Water, Kombucha Culture (Filtered Water, Black Tea Extract, Green Tea Extract, Natural Flavor), Filtered Water, Cane Sugar, Ginger Extract, Bacillus coagulans LactoSpore MTCC 5856, Black Tea Extract, Black Tea Essence, Caffeine (Green Coffee Bean Extract), Green Tea Extract, Stevia Leaf Extract
    K7; Lavender and Melon-flavored Raw Kombucha. Ingredients: Sparkling Water, Kombucha Culture (Filtered Water, Black Tea Extract, Green Tea Extract, Natural Flavor), Filtered Water, Cane Sugar, Bacillus coagulans LactoSpore MTCC 5856, Ginger Extract, Lavender Flavor, Watermelon Flavor, Black Tea Extract, Black Tea Essence, Caffeine (Green Coffee Bean Extract), Fruit and Vegetable Juice For Color, Green Tea Extract, Stevia Leaf Extract
    K8; Pineapple and Peach-flavored Raw Kombucha. Ingredients: Sparkling Water, Kombucha Culture (Filtered Water, Black Tea Extract, Green Tea Extract, Natural Flavor), Filtered Water, Cane Sugar, Bacillus coagulans LactoSpore MTCC 5856, Ginger Extract, Pineapple Flavor, Peach Flavor, Black Tea Extract, Black Tea Essence, Caffeine (Green Coffee Bean Extract), Green Tea Extract, Stevia Leaf Extract
  - Commercial fruit juice samples
    J1; Pressed Apple Juice. Ingredients: Apple Juice, Antioxidant (Ascorbic Acid)
    J2; Smooth Orange Juice. Ingredients: 100% Pure-squeezed Pasteurized Orange Juice
  - Commercial alcohol-free beer samples
    AFB1; Alcohol-free Dutch lager. Ingredients: Natural Mineral Water, Barley Malt, Wheat, Hops
    AFB2; Alcohol-free Danish Lager. Ingredients: Water, Malted Barley, Carbon Dioxide, Natural Flavoring, Hops, Hop Flavoring
- *Certified Reference Materials (CRMs)*—For this validation, K-ETOH was tested with the following certified reference materials
  Cerilliant E-037; Ethanol-80, 0.10%ABV (v/v)
  Cerilliant E-038; Ethanol-100, 0.13%ABV (v/v)
  Cerilliant E-041; Ethanol-150, 0.19%ABV (v/v)
  Cerilliant E-039; Ethanol-200, 0.25%ABV (v/v)
  Cerilliant E-044; Ethanol-40, 0.51%ABV (v/v)
  BCR651–beer at 0.505%ABV (v/v)
  BCR652–beer at 0.051%ABV (v/v)
- *Expression of results—*The concentration of ethanol is expressed in percentage Alcohol by Volume (%ABV) throughout

## AOAC *Official Method* 2019.08 Ethanol concentration in Kombucha beverages  Enzymatic method  First Action 2019

[Applicable to Kombucha beverages, fruit juice, alcohol-free beer, and low-alcohol beer]

**Caution:** The general safety measures that apply to all chemical substances should be adhered to. For more information regarding the safe usage and handling of this product please refer to the associated SDS that is available from the manufacturer’s website.

### Principle

The quantification of ethanol requires two enzyme reactions; in the first reaction catalyzed by alcohol dehydrogenase (ADH), ethanol is oxidized to acetaldehyde by nicotinamide-adenine dinucleotide.

(1) $$\text{Ethanol}+{\text{NAD}}^{\text{+}}\overset{\text{(ADH)}}{\to }\text{Acetaldehyde}+\text{NADH}+{\text{H}}^{+}$$

However, since the equilibrium of reaction (1) lies in favor of ethanol and NAD^+^, a further reaction is required to “trap” the products. This is achieved by the quantitative oxidation of acetaldehyde to acetic acid in the presence of aldehyde dehydrogenase (Al-DH).

(2) $$\text{Acetaldehyde}+{\text{NAD}}^{\text{+}}+{\text{H}}_{\text{2}}\text{O}\overset{\text{(AI-DH)}}{\to }\text{Acetic acid+NADH+H+}$$

The amount of NADH formed in this reaction pathway is stoichiometric with twice the amount of ethanol. It is the NADH which is measured by the increase in absorbance at 340 nm.

### Equipment

- Volumetric flasks (50 mL and 100 mL)
- Disposable plastic cuvettes (1 cm light path, 3.0 mL)
- Micro-pipettors (e.g., Gilson Pipetman^®^ P20 and P100)
- Positive displacement pipettor (e.g., Eppendorf Multipette^®^ - with 5.0 mL Combitip^®^ and 25.0 mL Combitip^®^)
- Analytical balance. (e.g., Mettler Toledo XS204)
- UV-VIS Spectrophotometer set at 340 nm (e.g., Amersham Biosciences Ultrospec 4300 pro spectrophotometer)
- Vortex mixer (e.g., IKA^®^ Yellowline Test Tube Shaker TTS2).
- Filter papers (e.g., Whatman No. 1, 9 cm)
- Disposable cuvette caps

### Chemicals and Reagents

All reagents are supplied as part of Megazyme’s Ethanol Assay Kit (Catalogue no. K-ETOH, see www.megazyme.com for purchase). Five bottles are supplied in the test kit, along with instruction for preparation. All reagents are stable as indicated on the label or kit box.

- Bottle 1: Buffer (15 mL, pH 9.0) plus sodium azide (0.02% *w/v*) as a preservative. Use the contents of bottle 1 as supplied.
- Bottle 2: NAD^+^. Dissolve the contents of bottle 2 in 12.4 mL of distilled water (to avoid repetitive freeze/thaw cycles, divide into appropriately sized aliquots and store in polypropylene tubes).
- Bottle 3: Aldehyde dehydrogenase solution (3.25 mL). Use the contents of bottle 3 as supplied. Before opening for the first time, shake the bottle to remove any enzyme that may have settled on the rubber stopper. Subsequently, store the bottle in an upright position. Swirl the bottle to mix contents before use.
- Bottle 4: Alcohol dehydrogenase suspension (1.3 mL). Use the contents of bottle 4 as supplied. Before opening for the first time, shake the bottle to remove any enzyme that may have settled on the rubber stopper. Subsequently, store the bottle in an upright position. Swirl the bottle to mix contents before use.
- Bottle 5: Ethanol standard solution (5 mL, 5 mg/mL). Dilute 0.5 mL of the contents of bottle 5 to 50 mL with distilled water in a volumetric flask. Store in a well-sealed Duran^®^ bottle. When diluted, this solution is stable for 2 days at 4°C.This component is only analyzed where there is any doubt as to the performance of the kit or the performance of the analyst.

### Other Considerations

This test should not be carried out by anyone other than a trained and experienced laboratory analyst. The ethanol control provided by the manufacturer should be included with analysis if there is any doubt as to the performance of the assay or the performance of the analyst.

The assay is very sensitive for ethanol and therefore it is important to ensure as much as possible that the laboratory environment and air is free from ethanol contamination. In particular, the use of ethanol-based bench cleaning sprays should be avoided.

Cuvettes should be capped during the analysis to isolate the enzymatic reaction from the general laboratory environment. It is also important to run a blank with every set of samples in order to ensure that the reagents have not been contaminated by ethanol from the environment. Contamination of the reagents would be visible as a gradual increase in absorbance of the blank over time. Losses in sample handling and dilution can be identified by performing recovery experiments, i.e., by adding ethanol to the sample in the initial dilution steps.

As Kombucha is a “live” product, it is imperative to ensure that correct sample handling is applied. Samples should be stored frozen and the user should not sample repeatedly from the same vial or container.

Kombucha samples used in this validation study were split into aliquots of approximately 10 mL upon receipt. These aliquots were stored frozen at -20°C. Aliquots were thawed and used on the same day in order to ensure that there could be no increase in ethanol concentration as a result of handling.

### Preparation of Test Materials

- Filter approximately 2 mL of sample solution using a 0.2-micron filter to remove live microorganisms that may be present in the sample.
- Mix the solution using a vortex mixer for approximately 30 s to remove any residual gas.
- Dilute the samples using distilled water to a concentration suitable for analysis. For Kombucha samples containing 0.063%ABV or more, a 200-fold dilution of sample is suitable. Add 0.5 mL of sample to a 100 mL volumetric flask and make to volume with distilled water. For samples containing ethanol at a level <0.063%, the dilution may be reduced accordingly (e.g., 20-fold dilution). Dilution troubleshooting is summarized in Table **2019.08A**.

**Table 2019.08A.** Summary of suggested sample preparation recommendations based on absorbance values achieved using the standard procedure (200-fold dilution of sample)

**Absorbance value achieved (A2-A1)**	**Recommendation**
<0.1	Create a more concentrated extract to yield an absorbance value between 0.1 and 1.2 (e.g., repeat the sample preparation example using a 20-fold dilution)
>1.2	Create a more dilute extract to yield an absorbance value between 0.1 and 1.2 (e.g., further dilute the prepared sample 10-fold to give a 2,000-fold final dilution)

### F. Assay Procedure

This procedure is summarized in Table 2019.08B.

- Set the spectrophotometer to read absorbance at 340 nm.
- Blank the spectrophotometer against air or water.
- Prepare a blank cuvette by addition of the following reagents to a 3 mL UV cuvette:
  2.1 mL of distilled water
  0.2 mL of Bottle 1 (Buffer)
  0.2 mL of Bottle 2 (NAD dissolved as described)
  0.05 mL of Bottle 3 (Aldehyde dehydrogenase)
  Cap the cuvette and mix by gentle inversion.
- Prepare a sample cuvette by addition of the following reagents to a 3 mL UV cuvette:
  2.0 mL of distilled water
  0.2 mL of Bottle 1 (Buffer)
  0.2 mL of Bottle 2 (NAD dissolved as described)
  0.05 mL of Bottle 3 (Aldehyde dehydrogenase)
  0.1 mL of sample (prepared as described)
  Cap the cuvette and mix by gentle inversion.
  Prepare in duplicate.
- After 2 min at room temperature (15–25°C) read the absorbances of the blank and then samples (A1).
- Remove the cuvette caps, taking care to avoid spillage of liquid.
- Start the reaction by addition of 0.02 mL of Bottle 4 (Alcohol dehydrogenase). Cap the cuvettes after addition and mix by gentle inversion.
- After 10 min at room temperature (15–25°C) read the absorbance of the blank and samples (A2).
- Ensure that the reaction has terminated by reading the absorbance of the blank and sample after a further 1 min at room temperature (15–25°C). If the absorbance value has increased at this time, continue to read at 1-min intervals until the rate of increase remains constant over a 5-min period. If this “creep” rate is greater for the sample than for the blank, extrapolate the sample absorbances back to the time of addition of Bottle 4. This can be simplified using the Megazyme MegaCalc tool for creep calculation (available on the manufacturer’s website).

**Table 2019.08B.** Summary of enzymatic determination reaction procedure

Wavelength	340 nm	
Cuvette	1 cm light path (glass or plastic with cap)	
Temperature	Room temperature (15–25°C)	
Final volume	2.57 mL	
Sample solution	0.25–12 µg of ethanol per cuvette	

Blank the spectrophotometer against air (without a cuvette in the light path) or against water.		

Pipette into cuvettes	Blank	Sample
Distilled water (∼20°C)	2.10 mL	2.00 mL
Sample	–	0.10 mL
Solution 1 (buffer)	0.20 mL	0.20 mL
Solution 2 (NAD^+^)	0.20 mL	0.20 mL
Solution 3 (Aldehyde DH)	0.05 mL	0.05 mL
Cap cuvettes, mix by gentle inversion and read the absorbances of the solutions (A_1_) after approx. 2 min. Start the reaction by addition of:		
Suspension 4 (Alcohol DH)	0.02 mL	0.02 mL

Mix by inversion and read the absorbances of the solutions (A_2_) at the end of the reaction (approx. 10 min). If the reaction has not stopped after 10 min, continue to read the absorbances at 1-min intervals until the absorbances increase constantly over 5 min.

### Calculations

Determine the absorbance difference (A2–A1) for both blank and sample. Subtract the absorbance difference of the blank from the absorbance difference of the sample, thereby obtaining ΔA_ethanol_. The value of ΔA_ethanol_ should as a rule be in the range of 0.100–1.2 absorbance units to achieve sufficiently accurate results.

The concentration of ethanol can be calculated as follows:

$$\text{c}=\frac{\text{V×MW}}{\text{ε}\times \text{d}\times \text{v}\times 2}\;\times \Delta {\text{A}}_{\text{ethanol}}\times \text{F [mg/mL]}$$

*where:*

V = final volume [mL]

MW = molecular weight of ethanol [g/mol]

ε = extinction coefficient of NADH at 340 nm

= 6300 [l x mol-1 x cm-1]

d = light path [cm]

v = sample volume [mL]

2 = 2 moles of NADH produced for each mole of ethanol

F = dilution factor

It follows for ethanol in %ABV terms:

$$\text{\%ABV=}\frac{\text{Ethanol }[\text{mg/mL}]}{7.894}$$

where 7.894 = factor to convert g/L to %ABV, taking the density of pure ethanol to be 0.7894 g/mL. If the sample has been diluted during preparation, the result must be multiplied by the dilution factor, F.

## VALIDATION

### Planning

**Figure 1:**
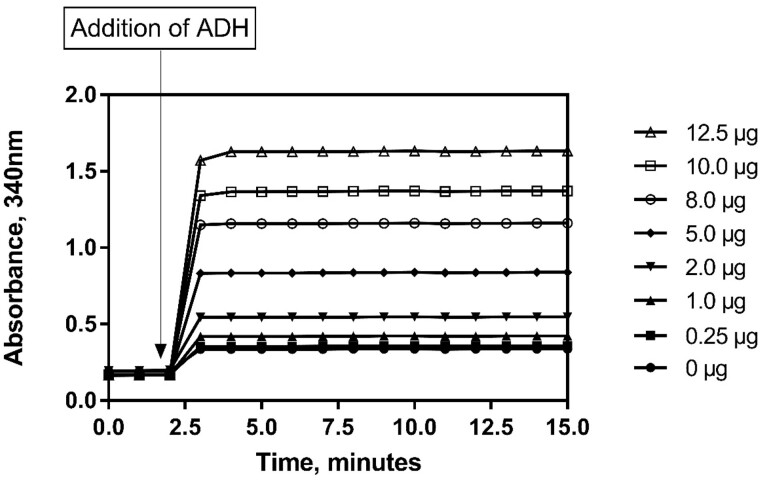
Reaction kinetic graph showing the time of reaction completion after addition of alcohol dehydrogenase (ADH) across a range of concentrations within the linear range of the assay.

The purpose of these experiments was to verify and validate the Ethanol Assay Kit (K-ETOH) for the analysis of ethanol in Kombucha. This single lab validation follows the AOAC recommendations as listed in Appendix K. This test requires the addition of one enzyme to begin the reaction. Absorbance (A2) was taken 10 min after the addition of the alcohol dehydrogenase (Bottle 4). All absorbances were read at 340 nm and at 20°C as unless stated otherwise. The reaction is complete within approximately 5 min, as shown in Figure 1, nonetheless it is recommended that the user measure absorbance 10 min after addition of the alcohol dehydrogenase.

### Performance Characteristics

Performance characteristics that are investigated within include: Working range, Selectivity, Limit of Detection (LOD), Limit of Quantification (LOQ), Trueness (*bias*), Precision (reproducibility and repeatability), Robustness, and Stability.

**Figure 2.**
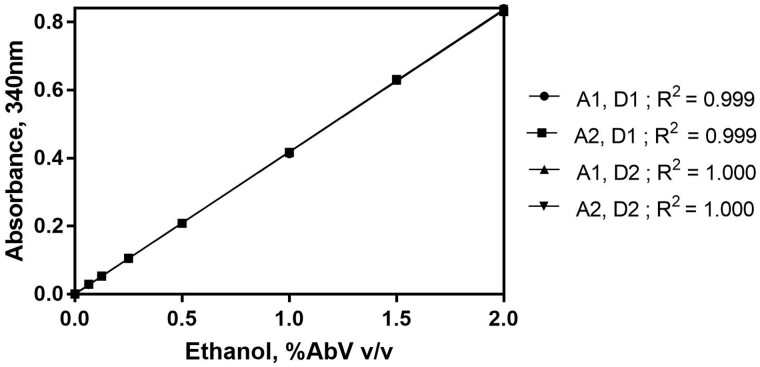
Examination of the linearity of the assay over a range of ethanol concentrations (0.05–2% AbV). Analysis carried out by two analysts over a period of two days. Key: A1 = Analyst 1; A2 = Analyst 1; D1 = Day; 1 D2 = Day 2.

**Figure 3.**
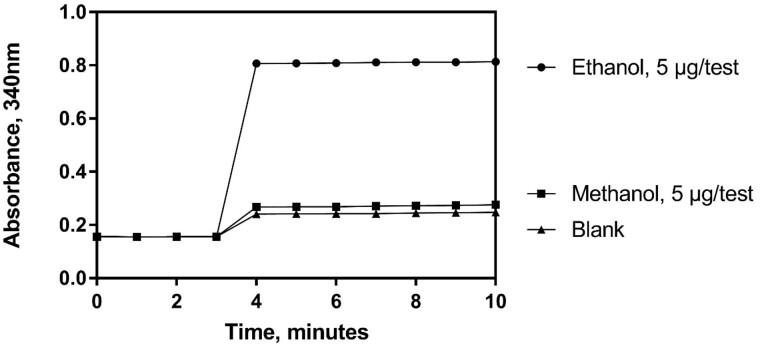
Examination of the conversion of methanol by the system, under the standard assay conditions.

**Figure 4.**
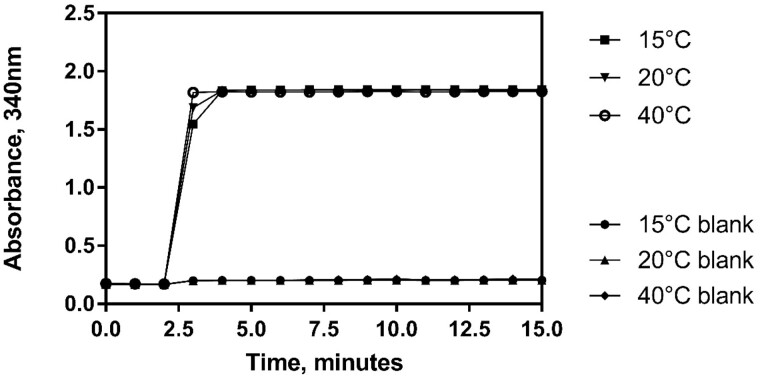
Examination of the robustness of the assay by adjusting the temperature at which the enzymatic determination reaction takes place. Assay carried out at 15°C, 20°C, and 40°C.

**Figure 5:**
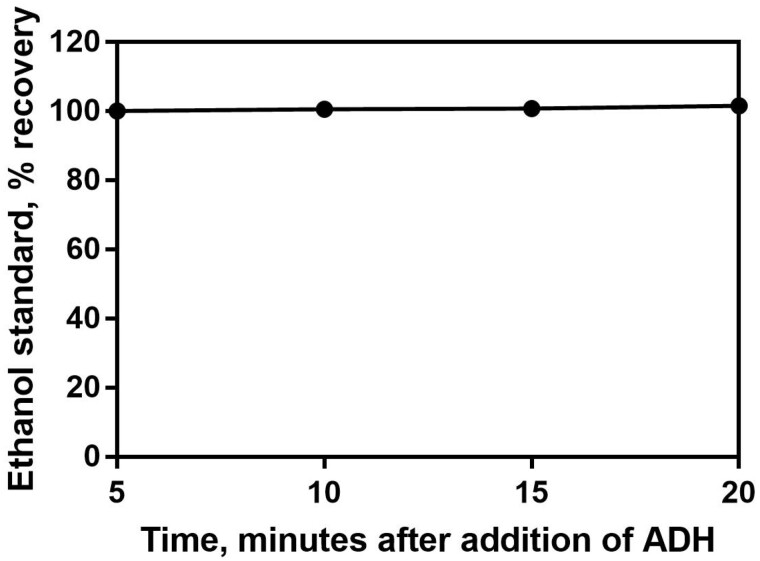
Examination of the robustness of the assay by adjusting the time at which the absorbance measurement is taken. Absorbance 340 nm measured at 5, 10 (recommended conditions for assay), 15, and 20 min after addition of alcohol dehydrogenase.

**Figure 6.**
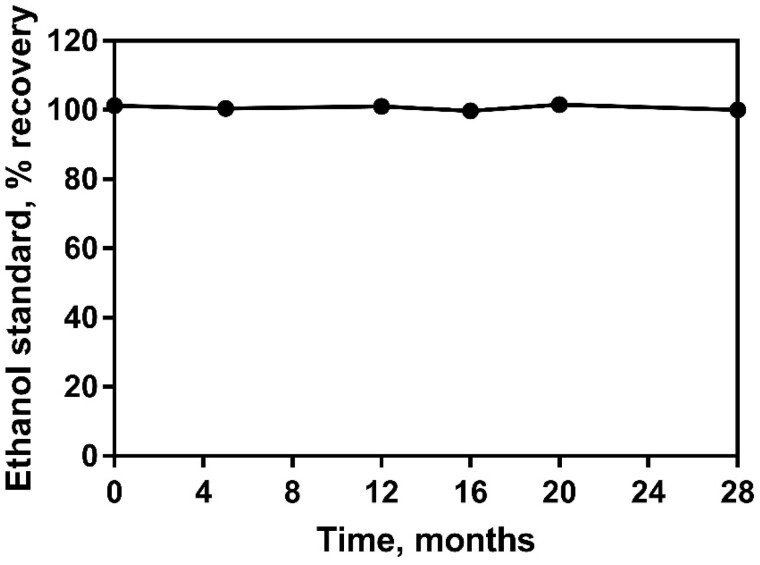
Stability of one set of kit components over a period of 28 months. The ethanol standard was assayed at the highest concentration (12.5 µg per cuvette) in order to thoroughly test the system for stability.

**Figure 7.**
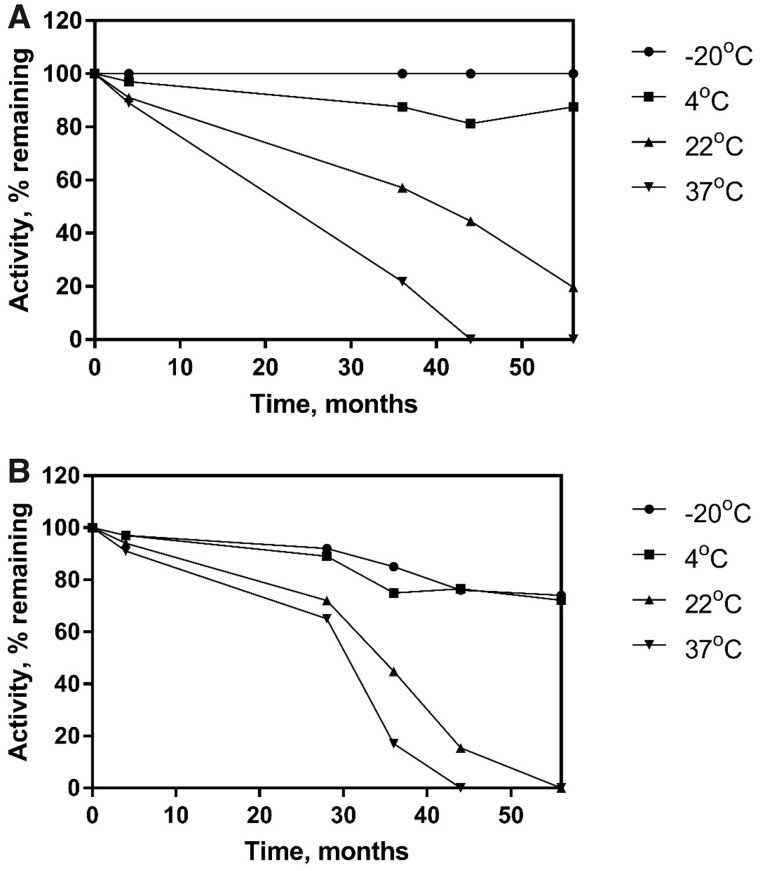
Stability of enzyme components (A) alcohol dehydrogenase and (B) aldehyde dehydrogenase over a period of 56 months. The enzymes were assayed for activity (U/mL) and then plotted as percentage activity remaining.

- Working Range
  The working range of the Ethanol Assay Kit was determined to be 0.25–12 µg of ethanol per test. A set of ethanol standards (prepared in-house) were tested across the stated linear range over a period of two days by two analysts and the results are depicted in Figure 2. The correlation factors (r^2^) are shown and prove linearity of the system across the stated range of concentrations with no variation between analysts. The repeatability of the system for both standards and samples is outlined in Precision.
  For samples treated as per the method outlined in this report (i.e., 0.5 mL of kombucha + 99.5 mL distilled water) the working range of 0.25–12 µg of ethanol per cuvette equates to a concentration range of 0.5–24 mg/mL or 0.063–3.04%ABV in original sample when using the minimum assay volume of 0.1 mL.
  It is possible for the user to alter the dilution factor for the sample where a lower concentration of ethanol is present in the sample. A linear response is achieved by dilution (data not shown).
- Selectivity and interference
  Alcohols present in the distilled water and buffers used for the assay, or in the air, can result in increased blanks or in creep reactions. Precautionary actions described previously can limit negative environmental effects. The order of addition of reagents during the assay reduces the possible interference from aldehydes and ketones, as any reaction with acetaldehyde dehydrogenase is taken into account in the A1 value, which can then be subtracted from the A2 value.
  The effect of acetaldehyde (CAS 75-07-0) on the A1 value was investigated by the addition of acetaldehyde, at varying concentrations, to the reaction cuvette prior to the addition of alcohol dehydrogenase. Acetaldehyde was added over a range of 0.3–3000 µg per test. The data is shown in Table 1. It is clear from the results that ethanol content is still measurable in the presence of acetaldehyde up to a concentration of 30 µg per cuvette. For a kombucha sample diluted 200-fold as per the recommended procedure, this equates to an acetaldehyde concentration of 60 mg/mL in the original sample. In practice, it is highly unlikely that acetaldehyde or other aldehydes will be present in kombucha at levels anywhere near the experimentally determined level shown to cause interference (30 µg/test).
  It was also experimentally determined that sulphite (added as sodium sulphite, CAS 7757-83-7), when present at or above 0.3 µg per test, interferes with ethanol recoveries. For a kombucha sample treated as per the method described in this report, this equates to a sulphite concentration of 600 mg/L in the original sample. The data is shown in Table 2. It is possible to conclude that there should be no interference from sulphite in Kombucha samples as even in wine, where sulphite is commonly added as a preservative, sulphite appears up to a level of 400 mg/L.
  The effect of methanol (CAS 67-56-1) on the system was also evaluated. An assumption was made that methanol should never appear in a kombucha sample anywhere near the level at which ethanol appears. Therefore, methanol was tested alongside an ethanol standard at the same concentration (5 µg/cuvette) and the change in absorbance monitored under the standard assay conditions. Methanol is not converted due to the unfavorable K*m*-values of the enzymes used. The results are shown in Figure 3.
  The effect of acetic acid (CAS 64-19-7) on the assay was investigated by the addition of acetic acid, at varying levels, to the reaction cuvette. Acetic acid was formulated over a range of concentrations (0.5–50 g/L) and added directly to the reaction cuvette (0.1 mL per test; 0.05–5 mg/test). The data for recovery at the top of the range (2.5–5 mg/test) is shown in Table 3. It is clear from the results that ethanol content is still measurable in the presence of acetic acid up to a concentration of 5 mg per cuvette. An acetic acid content of 0.2-0.4 g per liter of wine is considered normal.
  The assay is optimized for ethanol, however quantitative conversion of n-propanol and n-butanol, if present, is also achieved (data not shown).
- LOD and LOQ
  The calculated limit of detection (LOD) and the calculated limit of quantification (LOQ) for the purpose of this report is based on the analysis of a low-level concentration standard that has been taken through the procedure as outlined in this document.
  The LOD is the lowest concentration of the analyte that can be detected by the method. LOD is calculated as 3 x s’_0_; where s’_0_ is the standard deviation of replicates (n=25) of the ΔA_ethanol_ readings for the lowest standard used in this validation (0.1%ABV). The LOQ is the lowest level at which the kit’s performance is acceptably repeatable. LOQ is calculated as kQ x s’_0_; where s’_0_ is the standard deviation of replicates (n=25) of the ΔA_ethanol_ readings for the lowest standard used in this validation (0.1%ABV). The IUPAC default value for kQ is 10. The data and calculation are not shown.
  For samples treated as per the procedure as outlined in this report the LOD (Using 0.1 mL of sample in the enzymatic determination reaction) is 0.0012%ABV and the LOQ (using 0.1 mL of sample in the enzymatic determination reaction) is 0.0041%ABV.
- Trueness (*Bias*)
  Comparison of the mean of the results (x) achieved with the Ethanol Assay Kit for suitable reference materials with specific reference values (xref) across the liner range of the assay. For this report, Relative Bias is calculated in per cent as: *b*(%) = x–xref/xref x 100. The reference materials for this analysis are Cerilliant aqueous ethanol standards ranging from 0.1–0.51%ABV (Table 4) and BCR beer standards at 0.505% ABV ethanol and 0.051% ABV ethanol (Table 5). Excellent results were achieved and the results for these reference materials are shown in Tables 4 and 5.
- Precision
  This report details the repeatability of the Ethanol Assay Kit, a measure of the variability in results, on different days and by different analysts, over an extended period of time.
**System suitability** using the procedure as outlined in this report. Five certified reference materials were analyzed in order to provide system repeatability data (i.e., the suitability of the system for analysis of the specified ethanol concentrations, disregarding any possible matrix or sample influence on repeatability). The experiments were carried out by two analysts over a period of three days. Each “Analysis” as described in the table refers to a separate dilution of reference material by the analyst (Table 4).
**Repeatability (RSD_r_)** using the procedure as outlined in this report. Four commercial Kombucha products with ethanol concentrations between 0.6–1.3%ABV were analyzed in order to provide sample repeatability data (i.e., the suitability of the system for analysis of the specified sample type). The experiments were carried out by two analysts over a period of three days. Each “Analysis” as described in the table refers to a separate dilution of sample by the analyst (Table 6).
- Recovery
  Matrix effects are minimized by the requirement to dilute Kombucha samples 200-fold (where the sample contains ethanol 0.1–2%ABV). Nonetheless, a variety of sample spiking experiments were carried out in order to investigate this. Samples were spiked with known quantities of ethanol prior to treatment for assay (i.e., prior to filtration and dilution). Recoveries were assessed by comparison to sample assays (without spike) and assay of ethanol spike (without sample). Four Kombucha samples (K1–K4) were analyzed by one analyst three times each over a period of two days. Table 7 contains recovery data, showing the percentage recovery of ethanol spike for the four Kombucha samples. Spiking of Kombucha samples resulted in good recoveries between 98.5–102%. At the direction of the AOAC Expert Review Panel the method authors obtained several Kombucha samples containing very low levels of ethanol (K5–K8), in order to show functionality of the method when analyzing samples closer to the method LOQ. It was necessary to significantly reduce the dilution required for analysis (20-fold dilution). Sample spikes were carried out to examine any possible matrix effects. Excellent values were achieved and the results for these samples are shown in Table 8.
  Additional recovery data was obtained for fruit juice samples, for reasons outlined previously. Two fruit juice samples and two alcohol-free beer samples were analyzed by an individual analyst over a period of two days. Spiking of fruit juice and beer samples, although surplus to requirements, resulted in good recoveries between 95.3–101.2% (Tables 9 and 10).
- Robustness
  Robustness experiments were carried out to show that the method does not suffer due to variations or adjustments to certain parameters. Occasionally the user may lack the capability to control certain factors during the test (e.g., temperature at which the assay is run or time at which the absorbance is measured). The assay was carried out as per the procedure using the Megazyme ethanol standard solution (Bottle 5) over a range of temperatures. Figure 4 shows that the results do not suffer when adjustments are made to the temperature at which the assay is carried out, results remain the same after incubation at 15°C, 20°C, and 40°C although there is a very small variation in the time of completion of the assay. The time to completion does not negatively affect the result of the assay as absorbance (A2) should be read at 10 min.
  The time at which absorbance measurement is taken was also evaluated for robustness. In general, the enzymatic determination reaction is complete within 10 min of addition of alcohol dehydrogenase (Bottle 4). The assay was carried out as per the procedure (at 20°C) using the Megazyme ethanol standard solution (Bottle 5). Figure 5 shows that the results do not suffer when adjustments are made to the time at which the absorbance measurement is made, results remain the same when absorbance values are taken at 5, 10 (recommended), 15, and 20 min.
- Stability
  The Ethanol Assay Kit is formulated by Megazyme with a two-year stability guarantee when the components are stored as described on the individual kit component label. Kit components may be provided with longer stability guarantees, the user can find this information on the product label (specific expiry date is stated on each component). Regular quality control testing is performed in the Megazyme QC laboratory. The functionality of one set of kit components over a period of two years can be seen in Figure 6. As the Ethanol Assay Kit has been commercially available for a number of years, the historical data available from the Quality Control laboratory shows that the stability of each component does not vary from batch to batch (data not shown).
  The nature of the enzyme kit components (Bottles 4 and 5, acetaldehyde dehydrogenase and alcohol dehydrogenase) is such that they are the most likely to display poor stability. Therefore, detailed stability studies were carried out on these two components, measuring enzyme activity. Enzyme storage stability at -20°C (recommended storage for aldehyde dehydrogenase), 4°C (recommended storage for alcohol dehydrogenase), 22°C, and 37°C is shown in Figure 7 (U/mL values are converted to % activity for the purposes of this report).

**Table 1.** Table showing recoveries of ethanol standard in the presence of various concentrations of acetaldehyde under standard assay conditions. Absorbance (A1 value) is included in this table to illustrate the point that acetaldehyde interference can be taken into account up to a certain level by removal of A1 value from A2 value

Acetaldehyde, µg/cuvette	A1	Expected ethanol, % ABV	Measured ethanol, % ABV	% recovery
0.3	0.169	0.63	0.658	103.87
3	0.336	0.63	0.651	102.80
30	1.869	0.63	0.634	100.02
300	3.209	0.63	−0.133	−20.93
3000	2.568	0.63	−0.279	−44.05

**Table 2.** Table showing recoveries of ethanol standard in the presence of various concentrations of sulphite under standard assay conditions

Sulphite, µg/cuvette	Expected ethanol, % ABV	Measured ethanol, % ABV	% recovery
0.006	0.19	0.195	102.63
0.015	0.19	0.196	102.91
0.03	0.19	0.196	103.21
0.06	0.19	0.194	102.26
0.15	0.19	0.190	100.21
0.3	0.19	0.186	97.64
1	0.19	0.168	88.19

**Table 3.** Table showing recovery of an ethanol standard in the presence of various concentrations of acetic acid under standard assay conditions

Acetic acid, mg/cuvette	Expected ethanol, % ABV	Measured ethanol, % ABV	Recovery, %
2.5	0.0063	0.0064	100.6
3	0.0063	0.0062	97.9
3.5	0.0063	0.0062	97.1
4	0.0063	0.0062	97.7
4.5	0.0063	0.0062	98.1
5	0.0063	0.0063	99.8

**Table 4.** Trueness (*bias*) and intermediate precision (RSD_r_) of the method. Cerilliant aqueous ethanol standards diluted 200-fold (as per method recommended for kombucha samples) and analyzed (triplicate dilutions) by two analysts over three days. Results are shown in % ABV

Sample	E-044	E-039	E-041	E-038	E-037
Ethanol, % ABV (xref)	0.51	0.25	0.19	0.13	0.10
n	18	18	18	18	18
Analyst 1, Day 1	0.502	0.252	0.185	0.122	0.099
Analyst 1, Day 1	0.500	0.252	0.187	0.122	0.101
Analyst 1, Day 1	0.495	0.251	0.187	0.124	0.100
Analyst 1, Day 2	0.501	0.247	0.192	0.125	0.101
Analyst 1, Day 2	0.496	0.249	0.184	0.125	0.101
Analyst 1, Day 2	0.495	0.251	0.184	0.125	0.100
Analyst 1, Day 3	0.506	0.251	0.184	0.129	0.105
Analyst 1, Day 3	0.499	0.249	0.185	0.128	0.104
Analyst 1, Day 3	0.506	0.252	0.183	0.129	0.104
Analyst 2, Day 1	0.510	0.251	0.182	0.125	0.105
Analyst 2, Day 1	0.503	0.249	0.190	0.127	0.099
Analyst 2, Day 1	0.497	0.248	0.189	0.125	0.099
Analyst 2, Day 2	0.509	0.247	0.185	0.125	0.103
Analyst 2, Day 2	0.508	0.251	0.185	0.123	0.111
Analyst 2, Day 2	0.515	0.250	0.189	0.130	0.099
Analyst 2, Day 3	0.512	0.249	0.188	0.129	0.100
Analyst 2, Day 3	0.516	0.251	0.187	0.128	0.107
Analyst 2, Day 3	0.513	0.242	0.184	0.126	0.101
**Mean (x)**	**0.5047**	**0.2497**	**0.1863**	**0.1259**	**0.1023**
**SDEV**	**0.0069**	**0.0026**	**0.0026**	**0.0025**	**0.0033**
**RSD_r_**	**1.37**	**1.03**	**1.42**	**1.96**	**3.26**
**b(%)**	−**0.40**	−**1.46**	−**1.97**	−**0.63**	**0.90**

**Table 5.** Trueness (*bias*) and precision (%CV) of the method. Certified reference materials BCR651 and BCR652 diluted 200-fold (as per method recommended for kombucha samples) and analyzed by one analyst. Results are shown in % ABV

Sample	BCR652	BCR651
Ethanol, % ABV (xref)	0.051	0.505
**n**	**6**	**6**
Analyst 1, Day 1	0.050	0.504
Analyst 1, Day 1	0.051	0.488
Analyst 1, Day 1	0.049	0.488
Analyst 1, Day 1	0.050	0.510
Analyst 1, Day 1	0.049	0.489
Analyst 1, Day 1	0.049	0.504
**Mean (x)**	**0.050**	**0.497**
**SDEV**	**0.0009**	**0.0099**
**%CV**	**1.80**	**2.00**
**b(%)**	−**2.54**	−**1.55**

**Table 6.** Repeatability (RSD_r_) for four commercial kombucha samples analysed by two analysts over a three-day period. Results are shown in % ABV

Sample	Analysis	Ethanol, % ABV	Mean	STDEV	RSDr
**K1**	Analyst 1, Day 1	1.18	1.18	0.02	1.27
	Analyst 2, Day 1	1.16			
	Analyst 1, Day 2	1.21			
	Analyst 2, Day 2	1.18			
	Analyst 1, Day 3	1.18			
	Analyst 2, Day 3	1.19			
**K2**	Analyst 1, Day 1	1.52	1.52	0.02	1.24
	Analyst 2, Day 1	1.49			
	Analyst 1, Day 2	1.55			
	Analyst 2, Day 2	1.51			
	Analyst 1, Day 3	1.51			
	Analyst 2, Day 3	1.52			
**K3**	Analyst 1, Day 1	1.13	1.13	0.01	0.65
	Analyst 2, Day 1	1.14			
	Analyst 1, Day 2	1.14			
	Analyst 2, Day 2	1.12			
	Analyst 1, Day 3	1.13			
	Analyst 2, Day 3	1.12			
**K4**	Analyst 1, Day 1	0.80	0.78	0.01	1.12
	Analyst 2, Day 1	0.78			
	Analyst 1, Day 2	0.79			
	Analyst 2, Day 2	0.77			
	Analyst 1, Day 3	0.79			
	Analyst 2, Day 3	0.78			

**Table 7.** Recovery data for four commercial kombucha samples spiked with certified reference materials containing a known amount of ethanol across a range of concentrations (0.253–0.507% ABV). Expected ethanol = Concentration of spike added and Measured Ethanol = Concentration of spike measured

	Sample + spike	Ethanol spike		
Sample	Total ethanol, % ABV	Expected ethanol, % ABV	Measured ethanol, % ABV	Recovery, %
**K1**	1.70	0.507	0.515	101.58
	1.55	0.380	0.374	98.33
	1.44	0.253	0.255	100.45
**K2**	2.02	0.507	0.502	99.03
	1.89	0.380	0.374	98.53
	1.78	0.253	0.256	101.17
**K3**	1.63	0.507	0.499	98.54
	1.52	0.380	0.387	101.92
	1.39	0.253	0.255	100.55
**K4**	1.28	0.507	0.502	99.03
	1.16	0.380	0.380	100.02
	1.04	0.253	0.257	101.27

**Table 8.** Recovery data for four low ethanol commercial kombucha samples spiked with a known amount of ethanol. Expected ethanol = Concentration of spike added and Measured Ethanol = Concentration of spike measured

	Expected Ethanol (% ABV)	Measured Ethanol (% ABV)		
Sample	Ethanol spike	Sample only	Ethanol spike	Recovery (%)
K5	0.0063	0.0140	0.0063	99.49
K6	0.0063	0.0098	0.0064	101.54
K7	0.0063	0.0563	0.0064	101.60
K8	0.0063	0.0608	0.0064	100.90

**Table 9.** Recovery data for two commercial fruit juice samples spiked with certified reference materials containing a known amount of ethanol across a range of concentrations (0.253–0.507% ABV). Expected ethanol = Concentration of spike added and Measured Ethanol = Concentration of spike measured)

	Ethanol spike		
Sample	Expected Ethanol, % ABV	Measured Ethanol, % ABV	Recovery, %
**J1**	0.507	0.513	101.16
	0.380	0.371	97.73
	0.253	0.242	95.66
**J2**	**0.507**	**0.502**	**99.04**
	**0.380**	**0.368**	**96.79**
	**0.253**	**0.250**	**98.67**

**Table 10.** Recovery data for two commercial alcohol-free beer samples spiked with certified reference materials containing a known amount of ethanol across a range of concentrations (0.253–0.507% ABV). Expected ethanol = Concentration of spike added and Measured Ethanol = Concentration of spike measured)

	Expected ethanol, % ABV	Measured ethanol, % ABV		
	Ethanol spike	Sample only	Ethanol spike	Recovery, %
**AFB 1**	0.507	0.133	0.511	100.78
	0.380	0.131	0.384	101.11
	0.253	0.130	0.267	105.24
**AFB 2**	0.507	0.034	0.485	95.80
	0.380	0.033	0.363	95.60
	0.253	0.033	0.242	95.38

## Discussion

The Single Lab Validation as outlined in this report proves that the Ethanol Assay Kit (K-ETOH) is fit for purpose and applicable for the measurement of ethanol in kombucha as required by the AOAC SMPR 2016.001.

Kombucha samples tested for this report required a 200-fold or a 20-fold dilution, however the dilution can be changed where required, at the discretion of the analyst. The test kit is user-friendly and the assay is complete within 10 min from the addition of alcohol dehydrogenase (Bottle 4).

This SLV included investigation into a variety of performance characteristics including Working range, Selectivity, Limit of Detection (LOD), Limit of Quantification (LOQ), Trueness (*bias*), Precision (system suitability and repeatability), Robustness, and Stability.

The assay was shown to be linear over a range of 0.25–12.0 µg per test. For Kombucha samples treated as per the method outlined in this report, the working range of 0.25–12 µg of ethanol per cuvette equates to a concentration range of 0.5–24 mg/mL or 0.063–3.04%ABV of ethanol in the original sample when using the minimum assay volume of 0.1 mL.

The method can be considered to be specific for ethanol in Kombucha, under the assumption that Kombucha should not contain the interferants described in this report at levels approaching those stated. The large dilution of Kombucha required for assay also serves to remove any other matrix interference that may cause issues with determination, such as pH or sample color.

The Limit of Detection and Limit of Quantification were determined to be 0.0012%ABV and 0.0041%ABV respectively. Trueness was tested using aqueous ethanol standard reference materials and the results were excellent across a range of concentrations. Experiments showed high precision for all reference materials tested (including low-alcohol and alcohol-free beer reference materials) and all Kombucha samples. Excellent recoveries of ethanol spike were achieved for all Kombucha samples tested (98.5–102%) and good recoveries were achieved for fruit juice samples and alcohol-free beer samples tested (95.3–101.2%).

Robustness testing included the examination of incubation temperature (15°C, 20°C, and 40°C) and time at which absorbance measurement is taken (5, 10, 15, and 20 min). No parameter investigated during robustness testing was found to influence the result in any way.

All kit components were shown to have at least 2 years stability when stored as recommended and both enzyme components (alcohol dehydrogenase and acetaldehyde dehydrogenase) exhibit excellent stability for at least 4 years when stored as recommended (−20°C and 4°C, respectively). Note that a significant capacity for loss of activity for both enzymes is provided for in the product formulation as both enzymes are supplied at double the U/mL concentration required for full functionality in the test.

## Conclusions

The method outlined in this document is a robust, quick and easy method for the measurement of ethanol in Kombucha. Our data suggests this method is also reliable for similar matrices, such as low-alcohol beer and fruit juice. The assay meets all requirements set out in in AOAC SMPR 2016.001.
